# Refractive Indices of Fluids Related to Alternative Refrigerants

**DOI:** 10.6028/jres.099.022

**Published:** 1994

**Authors:** Thomas J. Bruno, Marcelo A. Wood, Brian N. Hansen

**Affiliations:** National Institute of Standards and Technology, Boulder, CO 80303

**Keywords:** alternative refrigerants, brominated/chlorinated ethanes, ethenes, ethers, propanes, propynes, refractive index

## Abstract

As part of a comprehensive program to develop suitable methods of chemical analysis for alternative refrigerants and their products, we have compiled a database of spectral, chromatographic, and physical property data that can aid in compound identification. As a small part of this effort, we have measured the refractive indices of a number of such fluids for which data were unavailable. The measurements were performed on a commercially available, digital Abbe refractometer that was modified for the relatively low temperature measurements (0 °C to 20 °C) that are sometimes required with these samples.

## 1. Introduction

The threat of atmospheric ozone depletion has lead to a great deal of research in many laboratories worldwide to find suitable substitutes for the fully halogenated fluids. These fluids have been used for many years as refrigerants, propellants, and blowing/foaming agents. Since the production of many of the older fluids is being phased out by law in most industrialized nations in the near future, there is a pressing need to thoroughly characterize the most promising substitutes. The Thermophysics Division of NIST has been a major force in this effort, with comprehensive experimental and theoretical thermophysical properties research.

Along with the efforts in thermophysical properties, an effort in the chemical analysis of these materials was developed out of necessity. This need arose because it is clearly impossible to understand the thermophysics of fluids of unknown or unreliable purity. Numerous analytical methods [1–4] and devices [5–16] have been introduced, and a comprehensive database of analytical data has been compiled [17–23]. This database contains spectral, chromatographic, and physical property information that is of value in the identification and analysis of alternative refrigerant fluids and byproducts. In this respect, the database covers fluid reaction products and common impurities—in short, any material that might have to be identified and quantified as part of the thermophysical properties work.

One of the most valuable physical properties for the identification of a material is the refractive index *n*_D_. Because it can be readily determined with a relative expanded uncertainty of a few parts in 10 000 (coverage factor *k* =2) [24], it is very useful and reliable in providing confirmatory evidence of the identity of a compound, especially in the liquid state. A large number of the materials of interest in alternative refrigerant research are newly synthesized, and therefore published values of refractive indices are often unavailable. As part of our efforts to provide as complete an analytical database as possible, we have measured the indices of refraction of 23 fluids for which no data were available.

We wish to emphasize that not all of these fluids are considered potential refrigerants. Indeed, many of these materials are heavily chlorinated or brominated, and are thus unsuitable from an ozone depletion point of view. It is important, however, to have the capability to readily identify these materials, since they may occur as reaction/decomposition products, or perhaps as residual impurities in field installations.

## 2. Experimental

The measurement of the refractive index of liquid samples is most often done with either an immersion refractometer or an Abbe refractometer. The immersion type is the most accurate instrument for use with liquids, typically producing measurements having standard deviations of 0.000 03. It is usable for refractive indices in the range 1.32 to 1.54. This instrument is somewhat inconvenient to handle, however, and requires a relatively large sample (10 mL to 15 mL) that must be maintained at constant temperature. Moreover, it can sometimes be plagued by sample viscosity effects. The Abbe refractometer, on the other hand, is an easily-used laboratory instrument amenable to much smaller sample sizes, usually just a few drops [25]. The smaller sample size makes temperature control of the sample much easier. This refractometer can be used for refractive indices in the range 1.3 to 1.7. It produces measurements of somewhat larger uncertainty than the immersion refractometer, with typical experimental standard deviations of 0.0001 [25–28]. In addition, it is a bit more complicated in construction than the immersion instrument.

Because of the relatively small quantities available for most of our samples, we used a digital Abbe refractometer for the measurements reported in this work. Since many of the fluids required measurement at a lower temperature than the nearly universal 25 °C of most reported liquid measurements, some simple modifications to the standard commercial instrument were required. The Amici prisms of the refractometer were thermostatted with a circulating low temperature bath that used ethanol as the working fluid. In addition, the optical housing of the refractometer was purged with a gentle flow of dry nitrogen to prevent condensation on the interior optics of the instrument. Some areas of the instrument were provided with glass-wool insulation. This was done for temperature control and as an added precaution against condensation of ambient moisture on critical surfaces. The samples were generally cooled in an ice bath prior to their being placed on the lower prism. The temperatures of the samples were measured before and after the measurement of each refractive index. Temperature was measured with a thermistor located in the lower Amici prism and had an expanded uncertainty of 0.05 °C.

The samples that were used for these measurements were either obtained commercially or were synthesized in other laboratories, and were of the highest available purity. They were used without further purification.

## 3. Results

The refractive indices of the fluids that were measured in this study are provided in Table 1, along with their respective refrigerant code numbers [29] and the temperatures at which the measurements were taken. The fluids are divided into the following classifications: ethanes, ethenes, brominated ethanes, propanes, propynes, and ethers.

## 4. Discussion

The repeatability of the measurements reported in Table 1 was assessed by performing multiple measurements in a relatively short time period under the same instrumental conditions. In general, only very slight variations (on the order of 0.01%) were noted between replicate measurements for each sample. In order to assess the longer term stability and reliability of the data, a large number of measurements were performed on one sample: 1,2,2-trichloropentafluoropropane, R-215a. During the course of several hours, 21 measurements were taken at 20.0 °C, and 21 were taken at 25.0 °C. The lower Amici prism was cleaned after each measurement, and sample was reapplied to the surface. The results are shown in Table 2, where the quoted uncertainty is the expanded uncertainty with a coverage factor *k* = 2 based solely on the experimental standard deviation of the mean of the 21 measurements. Probability plots constructed from both sets of data were linear, indicating that the deviations that were measured were normally distributed. We think that this level of reproducibility (approximately 0.01%) is indicative of that of the data provided in Table 2. The data are therefore of sufficient reliability for qualitative identification purposes. The experimental standard deviations are small compared to typical differences in refractive index that one observes from fluid to fluid. Performing such a number of multiple measurements for all of the samples was impossible because of the very limited supply available for most of these fluids.

The combined standard uncertainty of the measurements was assessed by measuring the refractive indices of several halocarbons having well established values of *n*_D_, as reported in the literature. On the basis of these comparisons, we estimate the final relative expanded uncertainty of the measurements presented here at 0.02%.

## Figures and Tables

**Table 1:** Refractive indices of the fluids measured in this study

Code number	Fluid	*n*_D_	Temperature (°C)
R-121	1,1,2,2-tetrachlorofluorocthane	1.4487	20.0
R-122	1,1-difluoro-1,2,2-trichloroethane	1.3922	20.0
R-131	2-fluoro-1,1,2-trichloroethane	1.4396	20.0
R-131a	1-fluoro-1,1,2-trichlorocthane	1.4252	20.0

R-1112aB2	1,1,-dihromodifluoroethene	1.4489	20.0

R-142B1	2-bramo-1,1-difluoroethane	1.3871	20.0
R-113B2αβ	2-chloro-1,2-dihromo-1,1,2-trifluorocthane	1.4281	20.0
R-114B2	1,2-dibromotetrafluorocthane	1.3708	20.0
R-123B2	1,2-dibromo-1,1,2 trifluorocthane	13720	20.0
R-123B1α	1-bromo-2-chloro-1,1,2-trifluorocthane	1.3721	20.0
R-133aB1	2,2,2-trifluorocthyl bromide	13429	5.0

R-215a	1,2,2-trichloropentafluoropropane	1.3497	25.0
		1.3525	20.0
R-215ba	1,2,3-trichloropentafluoropropane	1.4570	20.0
R-216ba	1,2-dichiorohcxafluoropropane	1.3114	5.0
R-225ca	3,3-dichloro-1,1,1,2,2-pcntafIuoropropane	1.3248	20.0
R-225cb	1,3-dichloro-1,1,2,2,3-pentafluoropropane	1.3265	20.0
R-243db	2,3-dichloro-1,1,1-trifluoropropane	1.3677	20.0
R-253fb	3-chloro-1,1,1-trifluoropropane	1.3298	20.0
R-262da	2-chloro-1,3-difluoropropane	1.3810	20.0
R-216B2	1,2-dibromohexafluoropropane	1.3596	20.0

R-2240	3-chloro-1-propyne	1.4362	20.0

R-E150a	α,α-dichloromethyl methyl ether	1.4070	20.0
R-E270b	2-chtoroethyl methyl ether	1.4370	20.0
R-E280	2,2-dichloroethyl methyl ether	1.4165	5.0

**Table 2:** Results of extended index of refraction measurements taken for R-215a

Temperature, °C	*n*_D_
(20.0±0.05) °C:	1.3525±0.0001
(25.0±0.05) °C:	1.3497±0.0001
